# Bioorthogonal Janus microparticles for photothermal and chemo‐therapy

**DOI:** 10.1002/SMMD.20240038

**Published:** 2024-11-11

**Authors:** Qingfei Zhang, Gaizhen Kuang, Kai Chen, Miaoqing Zhao, Luoran Shang

**Affiliations:** ^1^ Wenzhou Institute University of Chinese Academy of Sciences Wenzhou China; ^2^ Department of Orthopedics Shanghai Changhai Hospital Naval Medical University Shanghai China; ^3^ Department of Pathology Shandong Cancer Hospital and Institute Shandong First Medical University and Shandong Academy of Medical Sciences Jinan China; ^4^ Shanghai Xuhui Central Hospital Zhongshan‐Xuhui Hospital, and the Shanghai Key Laboratory of Medical Epigenetics International Co‐laboratory of Medical Epigenetics and Metabolism (Ministry of Science and Technology) Institutes of Biomedical Sciences Fudan University Shanghai China

**Keywords:** bioorthogonal, janus microparticle, microfluidics, photothermal therapy, prodrug

## Abstract

Bioorthogonal chemistry, recognized as a highly efficient tool in chemical biology, has shown significant value in cancer treatment. The primary objective is to develop efficient delivery strategies to achieve enhanced bioorthogonal drug treatment for tumors. Here, Janus microparticles (JMs) loaded with cyclooctene‐modified doxorubicin prodrug (TCO‐DOX) and tetrazine‐modified indocyanine green (Tz‐ICG) triggers are reported. Besides activating TCO‐DOX, Tz‐ICG is also a photothermal agent used in photothermal therapy (PTT), enabling the simultaneous use of biorthogonal chemotherapy and PTT. Additionally, the DOX could be significantly reduced in systemic toxicity with the modification of cyclooctene. Thus, the developed drug‐carrying JMs system exhibits effective tumor cell killing in vitro and effectively inhibits tumor local progress and distant lung metastasis after postoperative treatment with good safety. These results demonstrate that the prepared JMs provide a paradigm for bioorthogonal prodrug activation and localized delivery, and hold great promise for cancer therapy as well as other related applications.

## Graphical abstract

Janus microparticles were fabricated from microfluidics for bioorthogonal activatable prodrug loading and activation as well as combination therapy. The designed system demonstrated satisfactory efficiency in killing tumor cells in vitro and suppressing tumor growth in vivo through bioorthogonal chemotherapy and photothermal therapy.

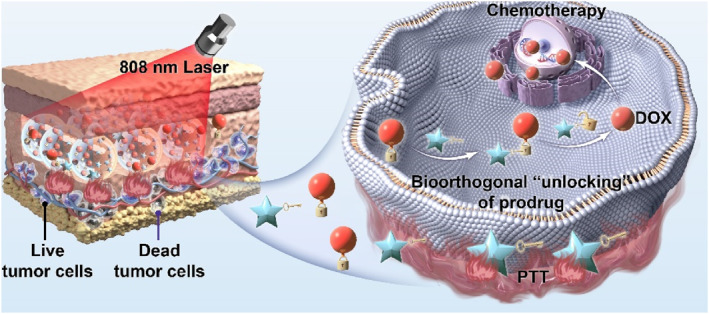

**Key points**

- Biorthogonal Janus microparticles (JMs) were fabricated from microfluidics as ideal platforms for biorthogonal prodrug delivery.
- Bioorthogonal prodrugs and activators were efficiently loaded into each side of the JMs.
- The JMs display effective anti‐tumor efficacy via the combination of bioorthogonal chemotherapy and photothermal therapy.

## 1 INTRODUCTION

Bioorthogonal chemistry involves reactions that can occur within living systems without affecting their intrinsic biochemistry.^1^, ^2^, ^3^ Bioorthogonal chemistry has revolutionized interdisciplinary research and opened new avenues for understanding, exploring, and manipulating biological systems with high precision and selectivity.^4^, ^5^, ^6^ Its applications in biomedicine, including imaging, diagnostics, and disease treatment, make bioorthogonal chemistry a potent tool in chemical biology.^7^, ^8^ In particular, bioorthogonal reactions can be used to develop precise biomolecular labeling, antibody‐drug conjugates, and prodrugs that are active against cancer.^9^, ^10^, ^11^ However, the limited bioavailability of small molecule prodrugs, coupled with their susceptibility to the method of administration, leads to inadequate drug concentration at the tumor site, consequently diminishing therapeutic effectiveness.^12^, ^13^, ^14^, ^15^ Besides, certain substrates engaged in bioorthogonal reactions, including transition metals, might unexpectedly manifest toxicity or immunogenicity within the biological system, imposing constraints on their clinical viability.^16^, ^17^ In addition, the complexity and challenges associated with designing and synthesizing bioorthogonal prodrugs and delivery systems have impeded their broad clinical applicability.^18^, ^19^, ^20^ Thus, there remains an anticipation for the advancement of a sophisticated system that integrates efficient drug loading with bioorthogonal activatable alongside convenient drug administration.

**FIGURE 1:**
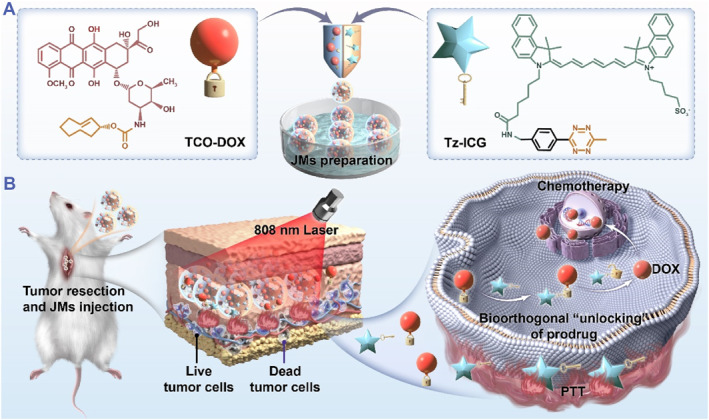
Schematic illustration of the fabrication of JMs and their utilization for tumor treatment. (A) Preparation of JM_ICG/DOX_ incorporating TCO‐DOX and Tz‐ICG through microfluidic electrospray techniques. (B) The therapeutic mechanism of JM_ICG/DOX_ involves the gradual release of TCO‐DOX and Tz‐ICG at the tumor resection site post‐injection. This release initiates a bioorthogonal reaction that subsequently activates the chemotherapeutic effects. Additionally, the JMs exhibit significant photothermal therapy (PTT) effects when exposed to 808 nm light irradiation, attributed to the intrinsic photothermal properties of Tz‐ICG. As a result, the JM_ICG/DOX_ demonstrated efficacy in eradicating residual cancer cells, thereby inhibiting both local tumor proliferation and distant metastasis through the integrated application of bioorthogonal chemotherapy and PTT.

In this paper, we fabricated biorthogonal Janus microparticles (JMs) from microfluidics for tumor treatment by synchronously realizing efficient drug loading, local administration, and bioorthogonal activation (Figure 1). Due to its high controllability, microfluidics allows the preparation of structurally tunable microparticles, which are widely used for disease treatment and drug delivery.^21^, ^22^, ^23^, ^24^, ^25^, ^26^, ^27^, ^28^, ^29^ Nevertheless, the application of microfluidic technology for developing carriers for bioorthogonal drug loading and activation is still in its infancy. Here, Janus microparticles (JM_ICG/DOX_) were designed to separately encapsulate cyclooctene‐modified doxorubicin (TCO‐DOX) prodrug and tetrazine‐modified indocyanine green (Tz‐ICG) activator in one platform. The implementation of prodrug modification strategies has the potential to significantly mitigate the systemic toxicity associated with DOX. It is important to note that the prodrug and activator are slowly released from JM_ICG/DOX_ during application at the tumor site, thereby facilitating a specific inverse electron‐demand Diels–Alder (IEDDA) reaction for DOX activation. The IEDDA reaction is generally classified as a bioorthogonal reaction that proceeds without the necessity of metal catalysis.^30^ By employing tetrazine structural components in Diels‐Alder cycloadditions with highly strained cycloalkenes, this reaction exhibits significant potential across a range of applications. Besides, because Tz‐ICG has intrinsic photothermal properties, the JM_ICG/DOX_ demonstrated efficient photothermal therapy (PTT) ability. Based on these features, the JM_ICG/DOX_ efficiently eliminated tumor cells in vitro and substantially suppressed post‐surgical tumors' local growth and distant metastases by combining bioorthogonal chemotherapy and PTT, with good safety. These findings highlighted the JMs as an ideal candidate for bioorthogonal drug delivery, also offering an effective and universal treatment system for various biomedical applications.

## 2 RESULTS AND DISCUSSION

**FIGURE 2:**
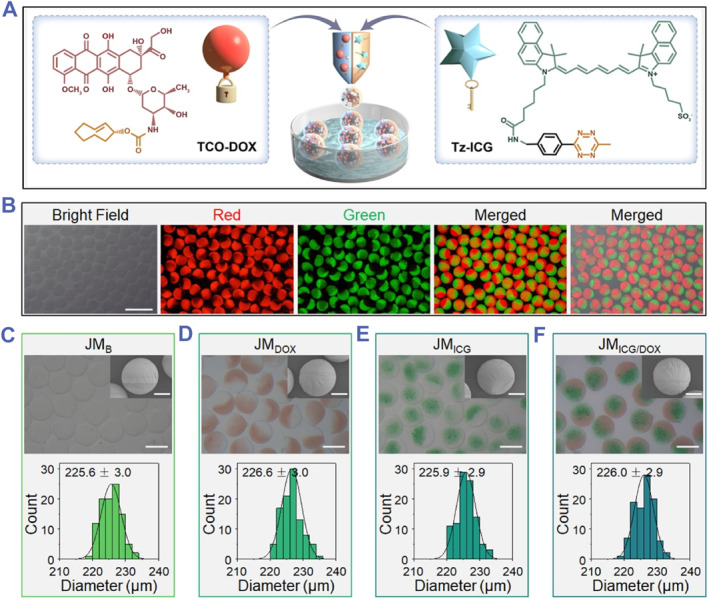
Preparation and characterization of JMs. (A) The diagram of JMs preparation. (B) Optical and fluorescent images of JMs loaded with red fluorescent particles and green fluorescent particles (Scale bar: 500 μm). (C–F) The optical images and scanning electron microscope (SEM) images of various JMs as well as their corresponding size distribution (Scale bars in optical images: 200 μm; in SEM images: 50 μm).

By using a glass capillary microfluidic installation, JMs were typically prepared through microfluidic electrospray, as shown in Figures 2A and S1. Two pregel solutions consisting of sodium alginate (SA), methacrylate gelatin (GelMA), and different drugs were pumped into each channel of the θ‐shaped capillary to generate Janus droplets under the electrical force. Thereafter, the droplets were gelated in a collection solution of CaCl_2_ and further crosslinked under ultraviolet light. By mixing with red and green fluorescent particles in the two pregel solutions, respectively, the two components of the JMs could be clearly distinguished through fluorescence microscopy (Figure 2B). Based on this, TCO‐DOX and Tz‐ICG molecules were loaded on each side of the JMs, denoted as JM_ICG/DOX._ Meanwhile, JMs containing only TCO‐DOX or Tz‐ICG were prepared and denoted as JM_DOX_ or JM_ICG_ (Figure 2C–F). As a control, JMs without drug loading were designated as JM_B_. Because of the distinctive colors of TCO‐DOX and Tz‐ICG, the JM_DOX_ and JM_ICG_ displayed red and green colors on the corresponding sides of the particles, respectively. In contrast, the drug‐free JM_B_ appeared colorless, whereas JM_ICG/DOX_ presented both colors. These characteristic colors were also reflected in tubes collected with various microparticles (Figure S2). Of note, according to previous reports, TCO‐DOX was synthesized by reacting DOX with (2E)‐TCO‐PNB (Figures S3–S6).^12^

**FIGURE 3:**
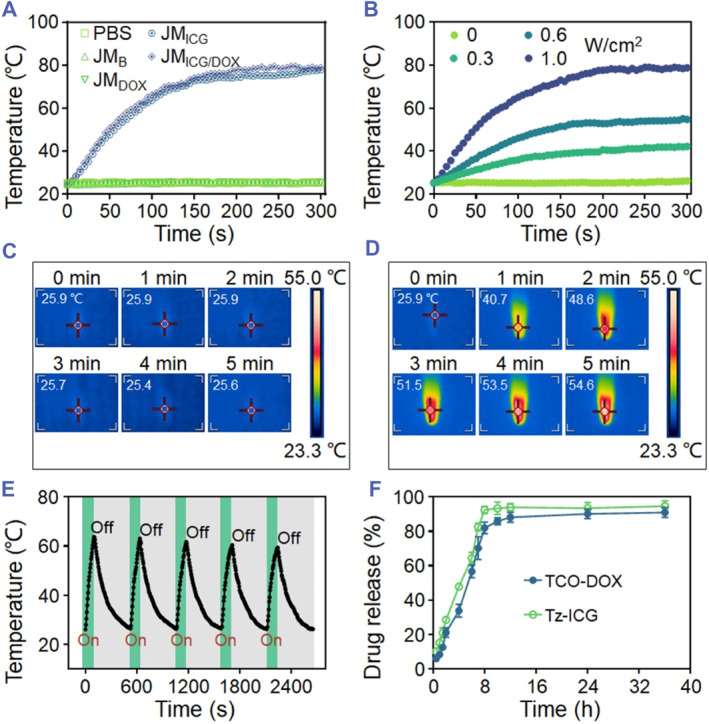
Photothermal effects of JMs. (A) Photothermal heating profiles of various JMs subjected to 808‐nm laser irradiation (1.0 W cm^−2^). (B) Photothermal heating profiles of JM_ICG/DOX_ under varying laser power densities. (C, D) Infrared thermal images of JM_B_ (C) and JM_ICG/DOX_ (D) under irradiation (0.6 W cm^−2^). (E) Temperature variation of JM_ICG/DOX_ subjected to five cycles of 808‐nm laser On/Off cycles (1.0 W cm^−2^, On for 100 s and Off for 420 s). (F) Drug release profiles of Tz‐ICG and TCO‐DOX.

ICG, an established organic dye approved by the Food and Drug Administration, has found extensive use in the field of PTT.^31^, ^32^ As a derivative of ICG, we reasoned that Tz‐ICG could also display photothermal performance. Thus, the photothermal property of the Tz‐ICG‐encysted microparticles was detected in detail. As displayed in Figure 3A, near‐infrared (NIR) laser irradiation caused a significant heating effect on JM_ICG_ and JM_ICG/DOX_ (both encapsulating Tz‐ICG), and after 5 min, a considerable temperature elevation was observed (Δ53.6°C for JM_ICG_ and Δ54.7°C for JM_ICG/DOX_). In contrast, negligible temperature changes were observed for JM_B_ and JM_DOX_ (without loading Tz‐ICG) under the same condition. Besides, increases in the power density of the laser were evidently associated with increased changes in the photothermal temperature of JM_ICG/DOX_ (Figure 3B). Using thermal images, the process of the photothermal heating process could be observed directly (Figures 3C,D and S7). Moreover, after undergoing five repetitive NIR‐On/Off cycles, JM_ICG/DOX_ demonstrated no significant temperature degradation (Figure 3E), underscoring the stability of the photothermal effect exhibited by these microparticles. These findings collectively substantiate the exceptional photothermal efficacy of the Tz‐ICG‐loaded microparticles, thereby establishing a foundational basis for future PTT research. Additionally, the drug‐release behaviors of JMs were investigated. The UV–vis–NIR spectrometer analysis revealed loading contents of 0.88 mg/g for TCO‐DOX and 1.29 mg/g for Tz‐ICG of JMs. Over an 8‐h period, 92.5% of Tz‐ICG and 82.1% of TCO‐DOX were released from JM_ICG/DOX_, with cumulative release amounts reaching 94.6% for Tz‐ICG and 91.0% for TCO‐DOX after 36 h (Figure 3F).

**FIGURE 4:**
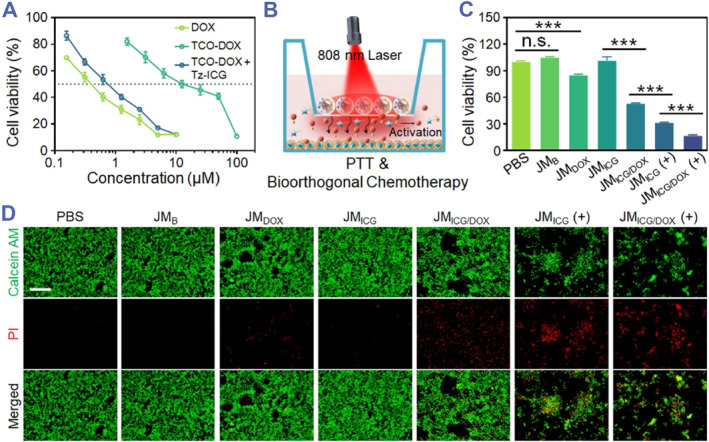
In vitro antitumor efficacy of JMs. (A) The CCK8 assay was performed on 4T1 cells following a 24‐h incubation period with various drugs. (B) Treatment diagram of JMs against 4T1 cells. (C) The CCK8 assay was conducted on 4T1 cells following a 24‐h incubation period with various JMs, both with and without irradiation. (D) Live/dead staining of 4T1 cells following a 24‐h incubation period with various JMs, with or without exposure to irradiation (Scale bar: 100 μm). (+) depicts treatments with irradiation (808 nm, 0.6 W cm^−2^, 5 min). All the cell experiments had three independent replicates (*n* = 3). Data are presented as the mean ± SD. n. s.: no significance, ***p < 0.001.

Afterward, the anti‐cancer activity of JM_ICG/DOX_ was performed. Firstly, Tz‐ICG was used to activate synthesized TCO‐DOX, and its cytotoxicity was examined before and after activation. As shown in Figure 4A, free DOX displayed strong cell‐killing effects and the half‐maximal inhibitory concentration (IC_50_) were 0.398 μM. By contrast, the efficacy of TCO‐DOX was evidently decreased with an IC_50_ of 12.6 μM due to the anticancer active site of amino in the DOX bonding with cyclooctene.^33^, ^34^ As expected, an inverse electron‐demand Diels‐Alder reaction (IEDDA) between TCO‐DOX and Tz‐ICG enhanced TCO‐DOX's therapeutic efficacy, and the IC_50_ value significantly decreased to 0.762 μM. It should be indicated that the Tz‐ICG had little cytotoxicity (Figure S8). Based on these findings, we demonstrated that the biorthogonal activation reaction is feasible and effective. Subsequently, the treatment effect of JM_ICG/DOX_ was evaluated in a 24‐well transwell plate system. After 5‐min irradiation, the JM_ICG/DOX_ was co‐incubated with 4T1 cells for another 4 h before removal (Figure 4B). After an additional 20‐h incubation period, the cell counting kit‐8 (CCK8) assay was used to assess the cell viability. As a result, neither JM_B_ nor JM_ICG_ affected cell proliferation, whereas JM_DOX_ exhibited a modest inhibitory effect on cancer cells (Figure 4C). However, JM_ICG/DOX_, when administered without irradiation, has shown significantly greater cytotoxicity than JM_DOX_ when it comes to cancer cells. Furthermore, the performance of JM_ICG_ with photothermal effects (+) was superior to that of its counterpart without irradiation. Notably, the JM_ICG/DOX_ (+)‐treated group demonstrated the highest inhibitory efficacy against 4T1 cells attributable to the combined effects of biorthogonal chemotherapy and PTT. Consistent results were observed through live/dead staining as illustrated in Figure 4D. The results demonstrate the superior anti‐cancer efficacy of JM_ICG/DOX_ in vitro due to the integration of PTT and biorthogonal‐activated chemotherapy.

**FIGURE 5:**
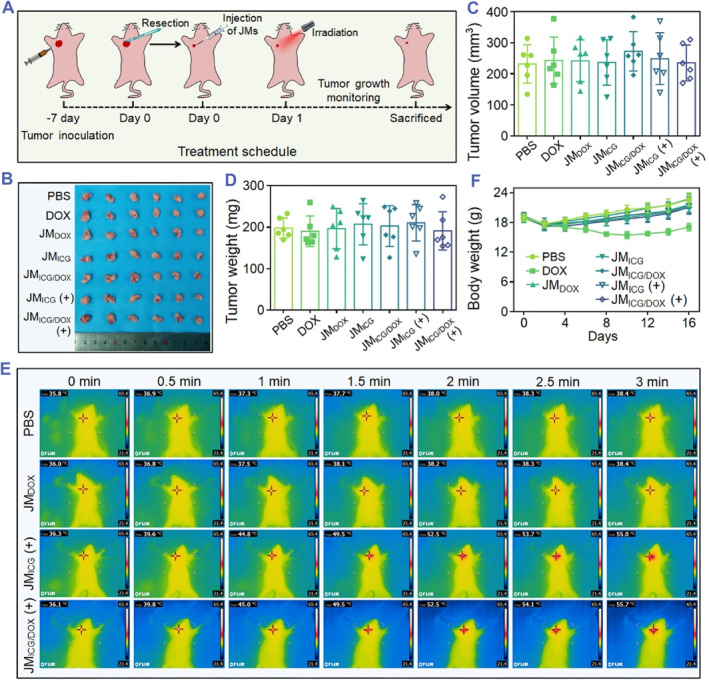
In vivo anti‐tumor procedure of JMs. (A) Schematic illustration of the tumor resection and JMs injection processes. (B–D) Photograph (B), volume (C), and weight (D) of the resected tumors. (E) Infrared thermal imaging of mice subjected to various treatments and exposed to irradiation for 3 min (0.6 W cm^−2^). (F) The body weight of mice during the 16‐day experiment. Each experimental group had 6 mice (*n* = 6).

Having confirmed the effective anti‐cancer ability of JM_ICG/DOX_ in vitro, the in vivo post‐surgical tumor eradication capacity of the JMs was further investigated. As shown in Figure 5A, we established an orthotopic breast tumor mice model with incomplete resection. Briefly, when the tumors grew up to approximately 240 mm^3^, Approximately 90% of tumor tissues were resected from each of the seven groups of mice (Figure S9). The image, size, and weight of the resected tumors were then measured, revealing evenly grouped mice in each group (Figures 5B–D). After the surgery, different types of JMs were injected into the excision site, and the in vivo photothermal effects of the corresponding treatment groups were investigated. Following a 3‐min exposure to NIR radiation, the temperature increase at the excision site injected with PBS and JM_DOX_ was merely ∼2.4°C, while the JM_ICG_ and JM_ICG/DOX_ groups achieved temperature elevation over 19°C (Figure 5E). Because the mice experienced the surgical operation, their body weight slightly decreased in the first 2 days (Figure 5F). Except for the group that received intravenous injections of DOX, the body weight of all the other treatment groups gradually recovered after surgery. Besides, during the 16‐day experiment, no significant pathological damage was found in the organs (Figure S10), implying the good safety of the JMs.

**FIGURE 6:**
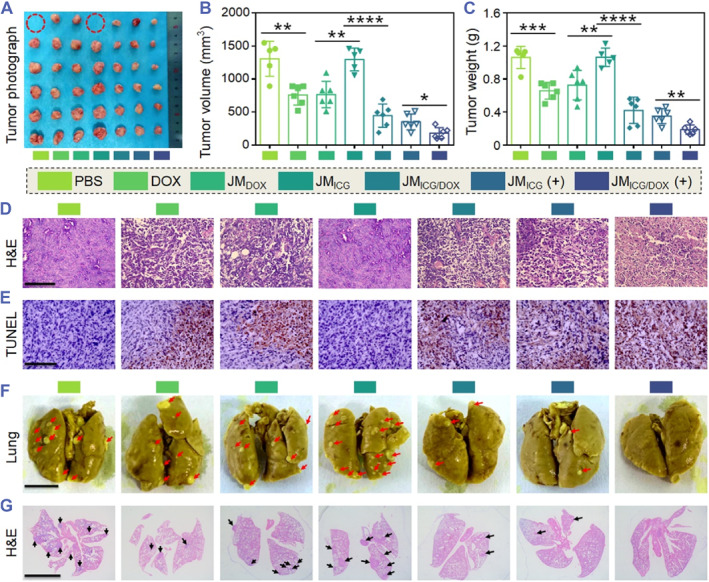
In vivo tumor inhibition and anti‐metastasis ability of the JMs. (A–C) Photograph (A), tumor volumes (B), and tumor weights (C) of 4T1‐tumor‐bearing mice after various treatments. The dotted red circles indicate the dead mice during the experiment. (D, E) H&E (D) and TUNEL (E) analyses of different groups (Scale bars: 100 μm). (F, G) Representative photographs (F) and H&E (G) staining of lungs with pulmonary metastatic nodules (indicated with red arrows and black arrows) after various treatments (Scale bars: 5 mm). Each experimental group had five mice (*n* = 6). Data are presented as the mean ± SD. *p < 0.05, **p < 0.01, ***p < 0.001.

The tumor inhibition and anti‐metastasis ability of JM_ICG/DOX_ were evaluated. Because of the uncontrolled tumor growth in both the control and JM_ICG_ groups, the tumor size and weight reached approximately 1300 mm^3^ and 1.0 g, respectively (Figure 6A–C). One mouse died in each of these two groups during the experiment due to the large tumor burden. Although the free DOX and JM_DOX_ treatments slightly retarded the tumor growth, the volume and weight of tumors were still large. In contrast, the JM_ICG/DOX_ group released Tz‐ICG and TCO‐DOX, and could further retard the growth of tumors through sustained bioorthogonal chemotherapy activation. Notably, the combined treatment of JM_ICG/DOX_ resulted in the most pronounced tumor inhibition, displaying the smallest tumor volume (approximately 180 mm³) and tumor weight (around 0.19 g), attributed to the synergistic effects of PTT and bioorthogonal chemotherapy. As anticipated, the JM_ICG/DOX_ (+)‐treated group displayed the most significant histological damage and the highest tumor tissue apoptosis as evidenced by hematoxylin and eosin (H&E) and terminal‐deoxynucleotidyl transferase‐mediated nick end labeling (TUNEL) analyses, respectively (Figure 6D,E). Moreover, tumor metastases to the lungs were identified through photographs and H&E staining (Figure 6F,G). The findings indicated that the group receiving JM_ICG/DOX_ (+) treatment exhibited the fewest metastatic tumor nodules (approximately 5 per lung) in comparison to the other groups (Figure S11), signifying the notable anti‐metastatic efficacy of JM_ICG/DOX_ (+). All these results revealed that the prepared JMs could efficiently suppress the growth of tumors and inhibit metastases via the synergistic action of PTT and bioorthogonal chemotherapy.

## 3 CONCLUSION

In summary, we have developed a formulation, JM_ICG/DOX_, which incorporates Tz‐ICG and TCO‐DOX by using droplet microfluidics to achieve synergistic effects in tumor PTT and chemotherapy. After application to the tumor resection site, the released TCO‐DOX can be activated by Tz‐ICG via IEDDA bioorthogonal reaction. This activation restores the cytotoxicity of DOX thereby facilitating effective chemotherapy. Besides, a PTT capability was demonstrated under laser irradiation of 808 nm for Tz‐ICG embedded in microcarriers. Thus, JM_ICG/DOX_ demonstrated synergistic effects of PTT and chemotherapy on tumor cells and inhibited tumor growth and distant metastases while displaying negligible systemic toxicity. These findings indicate that the JMs constitute an efficacious and universally applicable delivery platform for bioorthogonal chemotherapy, thereby exhibiting effective tumor therapy ability.

## 4 METHODS

*Materials:* Me‐tetrazine‐ICG (Tz‐ICG) and (E)‐Cyclooct‐2‐en‐1‐yl (4‐nitrophenyl) carbonate ((2E)‐TCO‐PNB) were obtained from Confluore Biotechnology Co. Ltd. Diisopropylethylamine (DIPEA), doxorubicin hydrochloride (DOX·HCl), and SA were bought from Macklin. The calcein‐AM/propidium iodide (PI) staining assay kit was bought from Meilunbio, Co., Ltd. Cell counting kit‐8 (CCK8) assay kit was brought from Beyotime Biotechnology Co. Ltd.

*Synthesis of TCO‐DOX:* Briefly, a mixture of 2E‐TCO‐PNB (15 mg, 0.05 mmol), DIPEA (64.5 mg, 0.50 mmol), and DOX·HCl (35 mg, 0.06 mmol) was stirred in the dark for 3 days at 30°C. 10 mL of water was added to the mixture and EtOAc (4 × 50 mL) was added afterward. After washing the combined organic phase with saturated NaHCO_3_, distilled water, and saturated NaCl solution, the organic phase was dried over anhydrous Na_2_SO_4_ for 2 hours. Finally, under reduced pressure, the solvent was evaporated leaving a residue that was purified using a dichloromethane‐methanol mixture (98:2) as the eluent yielding TCO‐DOX (20 mg, 57.5%).

*Preparation of JMs:* A glass microfluidic device was first fabricated, which contained two syringe needles, two round capillaries, an θ‐shaped capillary, and a glass slide. Each side of the θ‐shaped capillary was nested with one round capillary and then plated on the glass slide, followed by sealing with epoxy adhesives. The junction between the two round capillaries and needles was also sealed. A solution containing SA (2.0 wt%), GelMA (5.0 wt%), and TCO‐DOX (0.1 wt%) or red fluorescent particles (0.5 wt%) was prepared as pregel A. A solution containing SA (1.0 wt%), GelMA (2.0 wt%), and Tz‐ICG (0.2 wt%) or green fluorescent particles (0.5 wt%) was prepared as pregel B. Each of the pregel solutions was separately injected into each channel of the θ‐shaped capillary by syringe pumps to generate the Janus droplets (flow rate of 0.2 mL h^−1^, an electric field of 4 kV). The droplets were then solidified in a CaCl_2_ solution (2.0 wt%) and further crosslinked under ultraviolet light irradiation for 30 s to obtain the JMs. The morphology and size of the JMs were observed under a stereomicroscope and a SEM.

*Photothermal effect:* Various JMs (JM_B_, JM_DOX_, JM_ICG_, and JM_ICG/DOX_) underwent irradiation using a NIR light for 300 s, and temperature variations were monitored through a thermal imager (FLIR E5‐XT). Besides, we examined the temperature increase of JM_ICG/DOX_ under NIR light irradiation using different intensities (0, 0.3, 0.6, and 1.0 W cm^−2^). To assess photothermal stability, JM_ICG/DOX_ was exposed to NIR light (1.0 W cm^−2^) for 100 s (laser On) and allowed to cool naturally for 420 s (laser Off) for five cycles.

*Drug release study*: The JM_ICG/DOX_ microparticles were incubated in 10 mL of PBS at 37°C for 36 h in an oscillating incubator. 1 mL of the release medium was sampled and supplemented with the same volume of PBS solution at predetermined time points. In the collected medium, UV‐Vis‐NIR spectrophotometers were used to determine the concentrations of Tz‐ICG or TCO‐DOX.

In vitro *antitumor study:* 5 × 10^4 cells were placed into a 24‐well transwell plate and allowed to adhere for 12 h. Subsequently, various JMs were placed into the upper compartment plate. The concentration of TCO‐DOX in the JM_DOX_ or JM_ICG/DOX_ treatment groups was maintained at 30 μg. Thereafter, the JM_ICG_ or JM_ICG/DOX_ treatment groups were subjected to partial light irradiation for 5 min and incubated for another 4 h. The microparticles were subsequently removed, and the cells were incubated for an additional 20 h. Concurrently, the remaining groups were maintained in darkness for a total duration of 24 h. For live/dead staining, fluorescence microscopy was used to examine the status of these cells after they were treated with Calcein‐AM/PI. For the cell viability test, we measured the absorbance at 450 nm after the cells were incubated with the CCK8 reagent for 2 h.

*In vivo antitumor study:* In this study, we established tumors by subcutaneous inoculation of 1 × 10^6^ 4T1 cells into the right mammary region of mice, allowing the tumors to grow to approximately 240 mm^3^ in size. Subsequently, 90% of the tumor tissue in each mouse was surgically removed. To evaluate the photothermal performance of microparticles, mice received intratumor injections of PBS, JM_DOX_, JM_ICG_, or JM_ICG/DOX_ after surgery. Following the injections, the tumor sites were irradiated with a NIR light for 5 min and temperature variations were monitored using a thermal imager. Each group had six mice (*n* = 6).

To comprehensively evaluate the anti‐tumor effect of the developed JMs, the mice after surgery were randomly allocated into seven groups (*n* = 6): Saline, DOX·HCl (without irradiation), JM_DOX_ (containing 60 μg TCO‐DOX, without irradiation), JM_ICG_ (without irradiation), JM_ICG/DOX_ (containing 60 μg TCO‐DOX, without irradiation), JM_ICG_ (with irradiation, 0.6 W cm^−2^), and JM_ICG/DOX_ (containing 60 μg TCO‐DOX, with irradiation, 0.6 W cm^−2^). The mice received intravenous injections of DOX·HCl (3 mg kg^−1^), while in the other treatment groups, the JMs were injected into the tumor site after surgery.

Throughout the study, the mice were monitored every 2 days for their body weights and tumor volumes. After 16 days, we euthanized the mice, collected their primary organs and tumors, and preserved them by fixation in 4% (v/v) paraformaldehyde. For subsequent H&E and TUNEL staining, these specimens were sectioned into slices of 5 m thickness.

*Statistical analysis:* All statistical data are expressed as the mean ± SD. Statistical significance was calculated via unpaired Student's *t*‐tests. *p < 0.05, **p < 0.01, ***p < 0.001.

## AUTHOR CONTRIBUTIONS

Luoran Shang conceived the conceptualization and designed the experiment. Qingfei Zhang carried out the experiments and analyzed the data. Qingfei Zhang and Luoran Shang wrote the paper. Gaizhen Kuang, Kai Chen, and Miaoqing Zhao contributed to the scientific discussion of the article.

## CONFLICT OF INTEREST STATEMENT

Luoran Shang is an executive editor for *Smart Medicine* and was not involved in the editorial review or the decision to publish this article. All authors declare that there are no competing interests.

## ETHICS STATEMENT

The animal experiments were approved by the Animal Ethics Committee of the Wenzhou Institute, University of Chinese Academy of Sciences (approval WIUCAS23100401) and complied with the recommendations of the academy's animal research guidelines.

## Supporting information

Supporting Information S1

## Data Availability

The data that support the findings of this study are available from the corresponding author upon reasonable request.
