# Supplementary material for: Bioorthogonal Janus microparticles for photothermal and chemo‐therapy
Source: Smart Med. 2024 Nov 11;3(4):e20240038. doi: 10.1002/SMMD.20240038 (PMC11669771; doi:10.1002/SMMD.20240038)
Supplement: Supplementary file 1 — Supporting Information S1 [file SMMD-3-e20240038-s001.docx]

**Supplementary**

**Bioorthogonal Janus microparticles for photothermal and chemo-therapy**

Qingfei Zhang^1,#^, Gaizhen Kuang^1,#^, Kai Chen^2,^*, Miaoqing Zhao^3,^*, Luoran Shang^4,^*

^1^ Wenzhou Institute, University of Chinese Academy of Sciences, Wenzhou 325001, China

^2^ Department of Orthopedics, Shanghai Changhai Hospital, Naval Medical University, Shanghai 200433, China

^3^ Department of Pathology, Shandong Cancer Hospital and Institute, Shandong First Medical University and Shandong Academy of Medical Sciences, Jinan, China

^4^ Shanghai Xuhui Central Hospital, Zhongshan-Xuhui Hospital, and the Shanghai Key Laboratory of Medical Epigenetics, International Co-laboratory of Medical Epigenetics and Metabolism (Ministry of Science and Technology), Institutes of Biomedical Sciences, Fudan University, Shanghai 200032, China

# These authors contributed equally to this work.

Email: [luoranshang@fudan.edu.cn](mailto:luoranshang@fudan.edu.cn) (L.R. Shang); [zhaomqsd@163.com](mailto:zhaomqsd@163.com) (M.Q. Zhao); ch_kai@163.com (K. Chen)


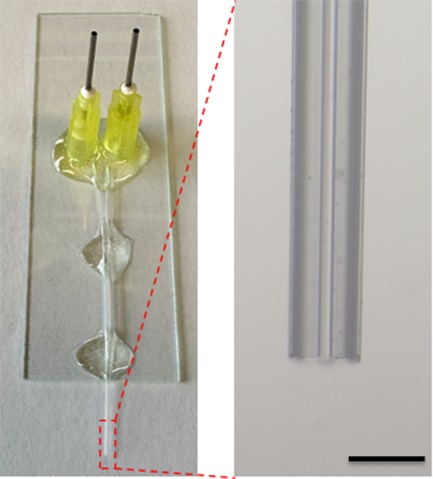


**Figure S1.** The glass capillary microfluidic device (Scale bar: 150 μm).


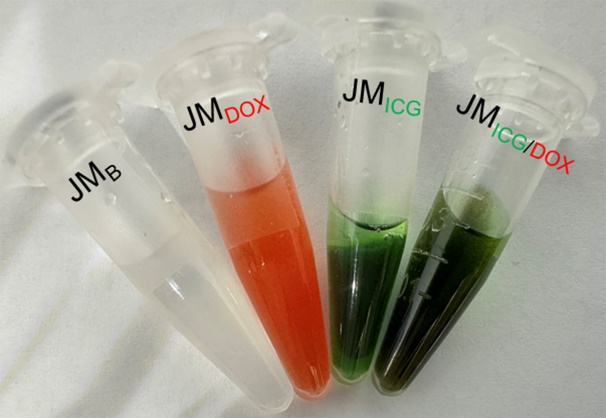


**Figure S2.** The photograph of JMs in tubes.

**
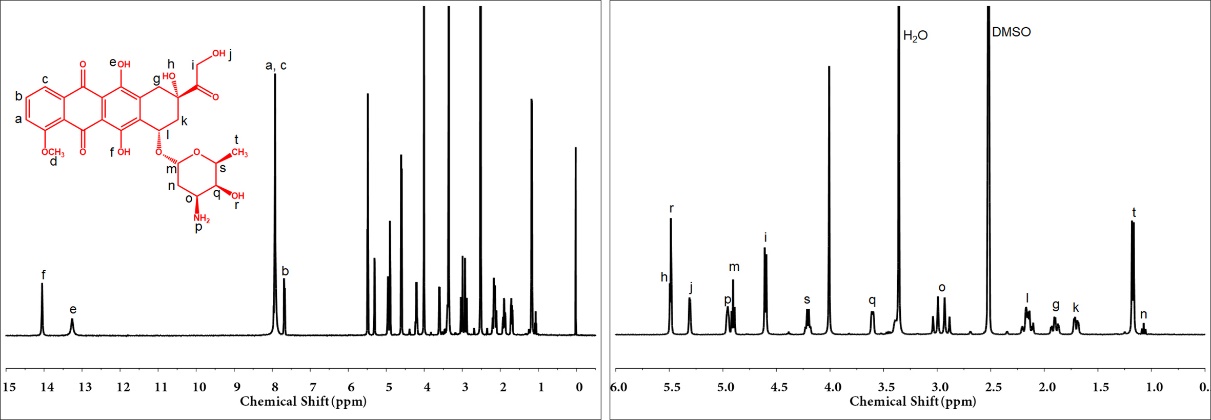
**

**Figure S3.** ^1^H NMR spectra of DOX in DMSO-d_6_.

**
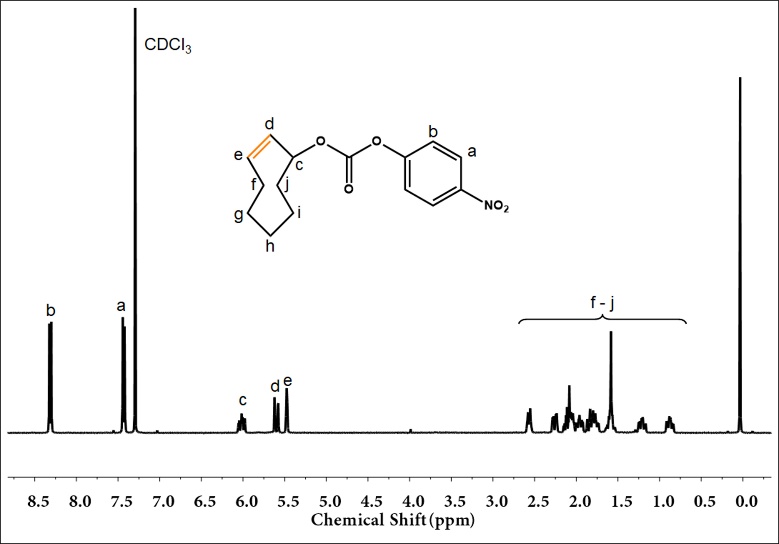
**

**Figure S4.** ^1^H NMR spectrum of (2E)-TCO-PNB in CDCl_3_.

**
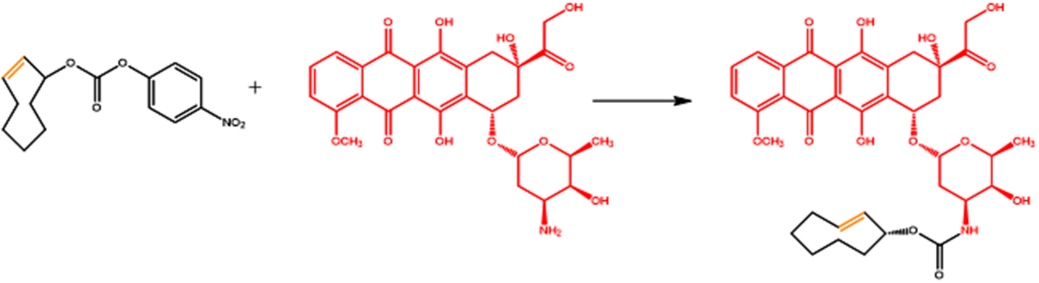
**

**Figure S5.** Synthesis pathway of TCO-DOX.

**
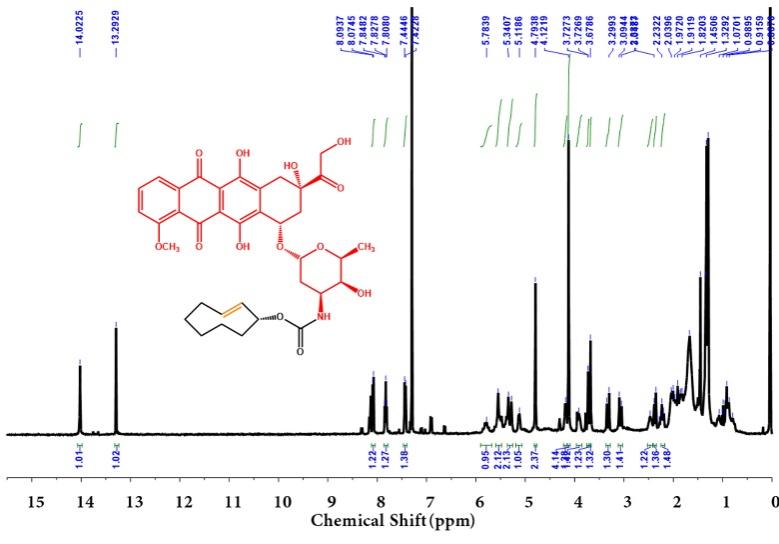
**

**Figure S6.** ^1^H NMR spectrum of TCO-DOX in CDCl_3_. δ (ppm): 13.99 (s, 1H), 13.26 (s, 1H), 8.06-8.04 (d, 1H), 7.79 (t, 1H), 7.40-7.38 (d, 1H), 5.86-5.64 (m, 1H), 5.51 (m, 1H), 5.30-5.24 (d, 2H), 5.12-5.04 (m, 1H), 4.76 (s, 2H), 4.14 (t, 1H), 4.08 (s, 4H), 3.87 (s, 1H), 3.69 (s, 1H), 3.64 (s, 1H), 3.31-3.26 (d, 1H), 3.30-3.26 (d, 1H), 3.05-3.01 (d, 1H), 2.43 (s, 1H), 2.31 (d, 1H).


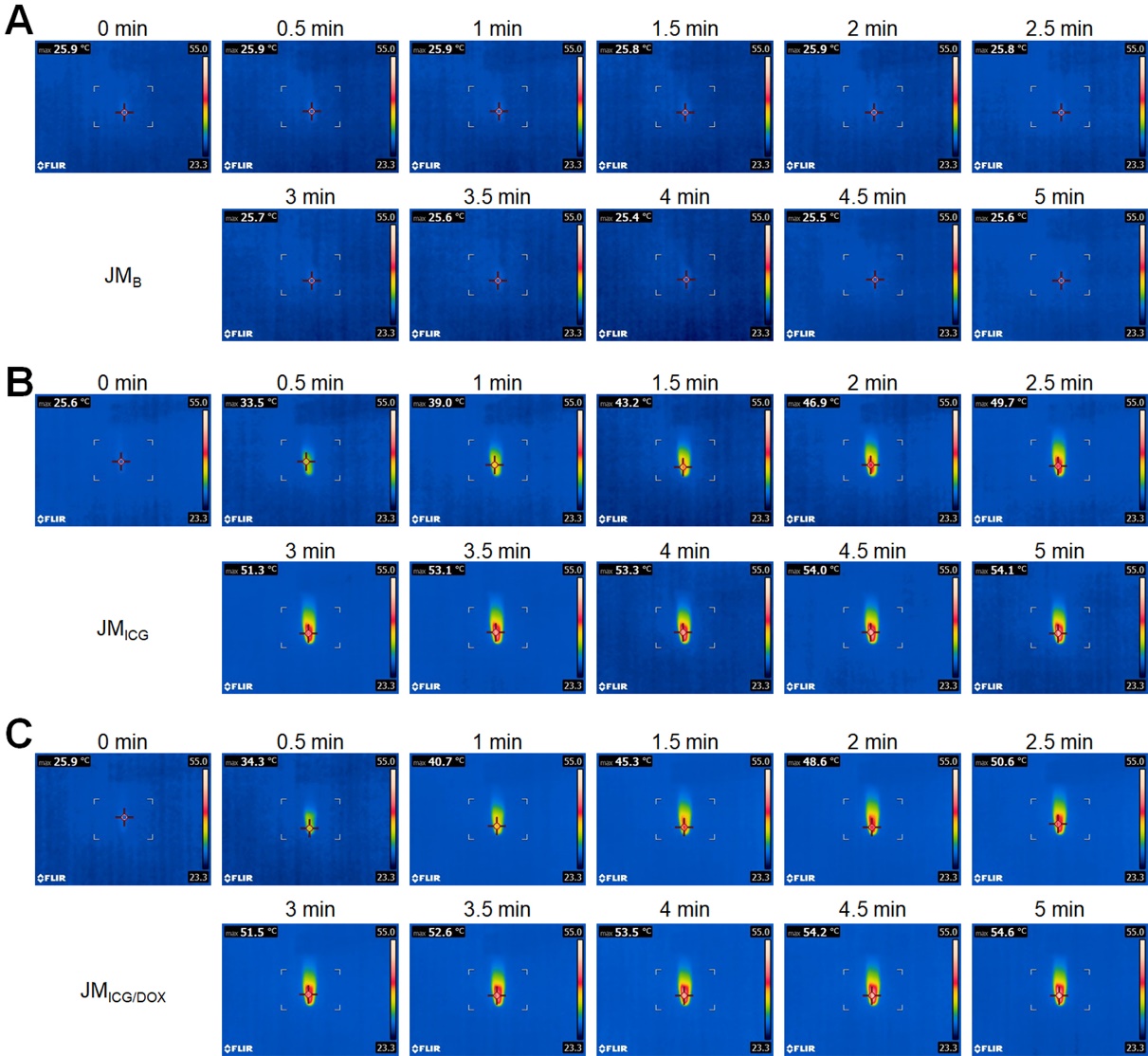


**Figure S7.** Infrared thermal images of JM_B_, JM_ICG_, and JM_ICG/DOX_ subjected to irradiation over varying time intervals (808 nm, 0.6 W cm^-1^).

**
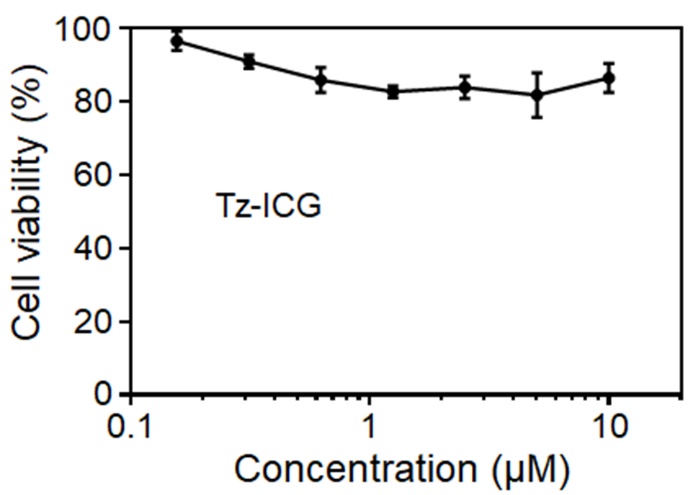
**

**Figure S8.** A CCK8 assay was performed on 4T1 cells following a 24-hour incubation period with Tz-ICG.

**
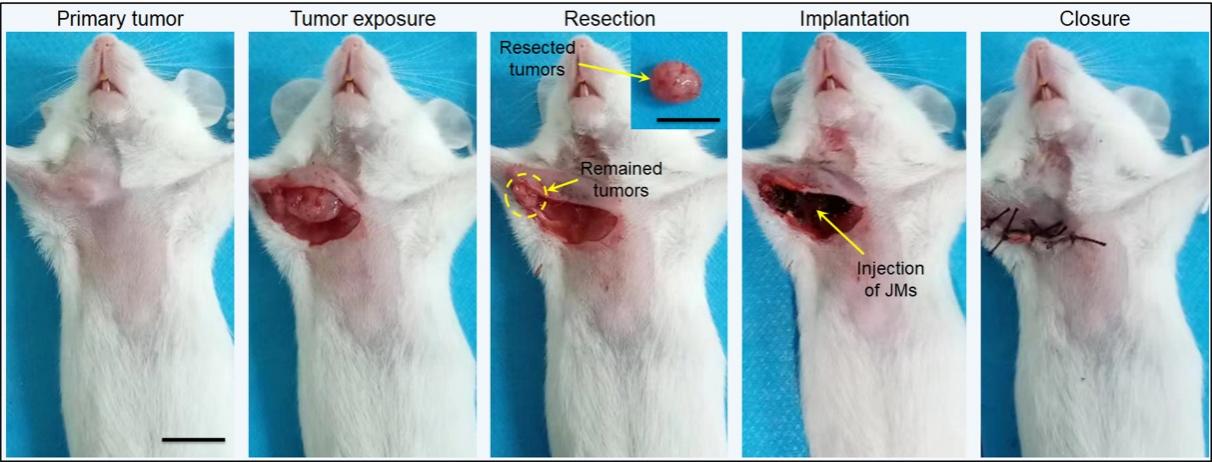
**

**Figure S9.** The processes of tumor resection and JMs injection (Scale bar: 1 cm).


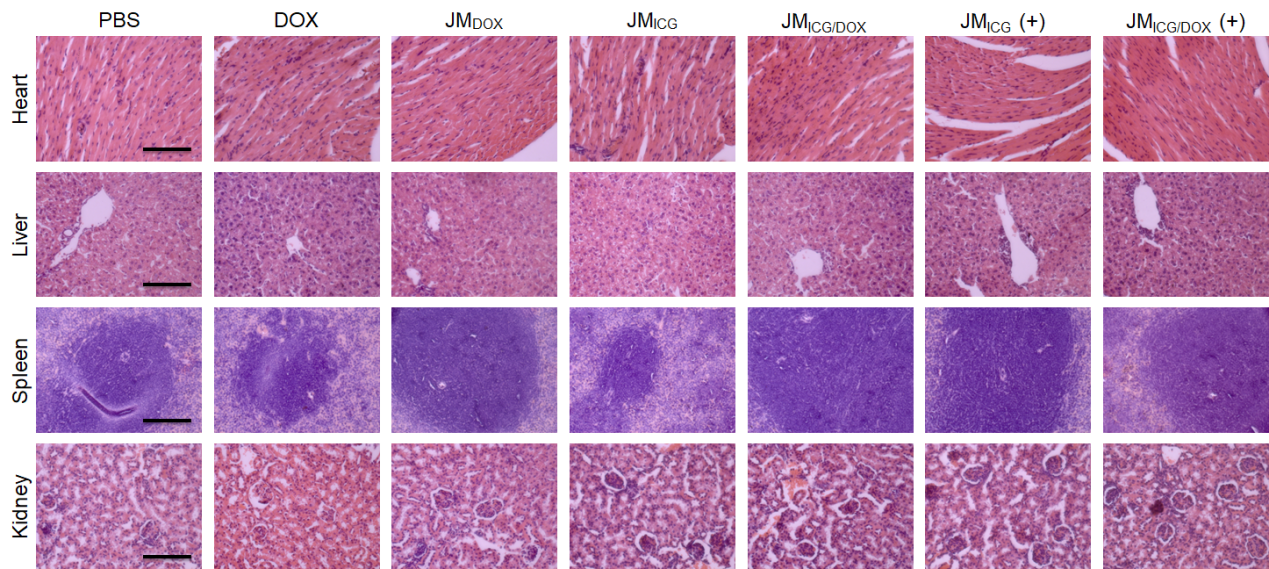


**Figure S10.** H&E staining of major organs (Scale bar: 100 μm).


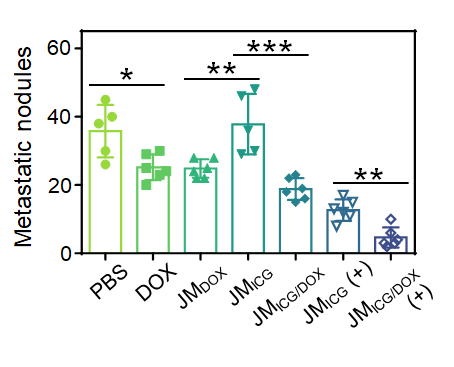


**Figure S11.** Quantification of the pulmonary metastatic nodules after different treatments.
